# Prevalence of hepatitis B virus infection and treatment eligibility in Lesotho, Southern Africa: a population-based cross-sectional study with case-based follow-up

**DOI:** 10.1136/bmjph-2024-001195

**Published:** 2024-11-29

**Authors:** Emmanuel Firima, Rameno Ntsoaki, Blaise Lukau, Mosa Tlahali, Lucia Gonzalez Fernandez, Molulela Manthabiseng, Mamoronts’ane P Sematle, Matumaole Bane, Makhebe Khomolishoele, Leisa Ikhetheleng, Lefokosane Retselisitsoe, Ravi Gupta, Stephen McCrosky, Tristan Lee, Frederique Chammartin, Maja Weisser, Niklaus D Labhardt, Alain Amstutz

**Affiliations:** 1 Division of Clinical Epidemiology, Department of Clinical Research, University Hospital Basel, University of Basel, Basel, Switzerland; 2 School of Medicine and Population Health, University of Sheffield, Division of Clinical Medicine, Sheffield, UK; 3 Butha Buthe Government Hospital, Butha Buthe, Lesotho; 4 SolidarMed, Partnerships for Health, Maseru, Lesotho; 5 Mokhotlong District Health Management Team, Mokhotlong, Lesotho; 6 Swiss Tropical and Public Health Institute, Allschwil, Switzerland; 7 Division of Infectious Diseases and Hospital Epidemiology, University Hospital Basel, Basel, Switzerland; 8 Ifakara Health Institute, Ifakara, Tanzania; 9 Population Health Sciences, Bristol Medical School, University of Bristol, Bristol, UK; 10 Oslo Center for Biostatistics and Epidemiology, Oslo University Hospital, Oslo, Norway

**Keywords:** Epidemiology, Public Health, Communicable Disease Control

## Abstract

**Background and aims:**

There is no data on hepatitis B virus (HBV) prevalence and treatment eligibility among the general population in Lesotho. We aimed to determine the prevalence of HBV infection in a large-scale cross-sectional survey among the general population in Lesotho, assess determinants of seropositivity, and evaluate treatment eligibility according to the 2024 WHO guidelines.

**Approach and results:**

We conducted a household-based, cross-sectional survey among participants≥10 years old in 120 randomly sampled village clusters in two districts. From participants screened positive for HBV surface antigen (HBsAg), we collected dried blood spots for HBV DNA measurement and referred the participants to health facilities for clinical assessment and treatment eligibility evaluation.

Out of 6709 participants screened, 6705 had a valid HBsAg test result (3509 (52.3%) female, median age 33 years (IQR: 20–53)), which was positive in 78 participants, yielding a prevalence of 1.2% (95% CI: 0.9 to 1.4). Being≥18 years old, male, living in urban areas, living with HIV, consuming tobacco and belonging to higher wealth index quintiles, were associated with increasing odds of HBV infection. Of the 78 participants with HBV infection, 62 (79.5%) linked to care. Among these, 25/62 (40.3%) were also living with HIV and 23/25 (92%) already taking antiretroviral treatment active against HBV. Among the remaining, 10/37 (27.0%) were eligible for antiviral treatment based on HBV DNA, Aspartate aminotransferase to Platelet Ratio Index or alanine aminotransferase levels.

**Conclusions:**

We observed a low prevalence of HBV infection among Basotho. Treatment eligibility was high mostly due to the presence of HIV co-infection. However, nearly one-third of HBV mono-infected participants were eligible for treatment, suggesting a testing and treatment gap in this population.

**WHAT IS ALREADY KNOWN ON THIS TOPIC**

- In Lesotho, all publicly available data on hepatitis B virus (HBV) prevalence and treatment eligibility are from individuals with HIV co-infection.

**WHAT THIS STUDY ADDS**

- We found a 1.2% prevalence of HBV infection in the general population, substantially higher in the pre-HBV vaccination age group. Using the 2024 WHO guidelines, overall treatment eligibility was high, with nearly one-third of HBV mono-infected participants being eligible to receive treatment.

**HOW THIS STUDY MIGHT AFFECT RESEARCH, PRACTICE OR POLICY**

- This study provides crucial public health information for Lesotho and will inform policy and planning in the country and the region to close the treatment gap for HBV. These findings highlight the difficulty in applying the WHO treatment eligibility guidelines in a setting such as Lesotho, where routine HBV viral load testing and FibroScan are not routinely available.

## Background

 Globally, about 2 billion people have been acutely infected with hepatitis B virus (HBV), and over 300 million people have a chronic infection.^1^,^3^ Currently, there is no cure for chronic HBV infection, and persons with chronic infection have a 15–40% lifetime risk of liver cirrhosis, liver failure or hepatocellular carcinoma.^4 5^ The WHO estimates that in 2015, 887 000 people died from hepatitis B-related liver disease, with low- and middle-income countries bearing the highest burden.^25^,^7^ In sub-Saharan Africa, an estimated 82 million people currently live with HBV.^3^

WHO has set an agenda for the global elimination of viral hepatitis as a public health threat by 2030.^8^ HBV vaccines are an effective tool to prevent and control HBV infection, offering nearly 100% protection against the virus.^9 10^ In the early 90s, WHO recommended the inclusion of the hepatitis B three-dose primary series in national immunisation schedules, with varying implementation by countries.^2^ Available data show that Lesotho introduced the hepatitis B vaccine in 2003.^11^ Furthermore, antiretroviral agents such as nucleoside/nucleotide reverse transcriptase inhibitors have the potential to alter the natural history of chronic hepatitis B_12_, and are recommended in patients with a co-infection with HIV and in HIV-negative individuals with HBV infection and signs of liver inflammation.^12 13^

However, only a few people are aware of their HBV infection status globally. The WHO reports that in 2016, 10.5% of individuals with HBV infection were diagnosed, and 17% of those diagnosed received antiviral treatment.^14^ Evidence on the number of people infected with HBV who meet treatment eligibility criteria in different global regions is scant.^5 6^ Published data indicate that in Africa, only 2% of people living with chronic HBV infection are diagnosed, and 0.1% taking treatment.^15^ In Lesotho, Southern Africa, few studies report on the prevalence of HBV infection, and if so, only among an HIV-co-infected population.^16 17^

Our objectives were to estimate the prevalence of HBV infection among the general population in Lesotho, assess the determinants for acquiring the infection and assess treatment eligibility among participants identified to have HBV infection according to the 2024 WHO guidelines.

## Method

### Design, setting and participants

**Figure 1:**
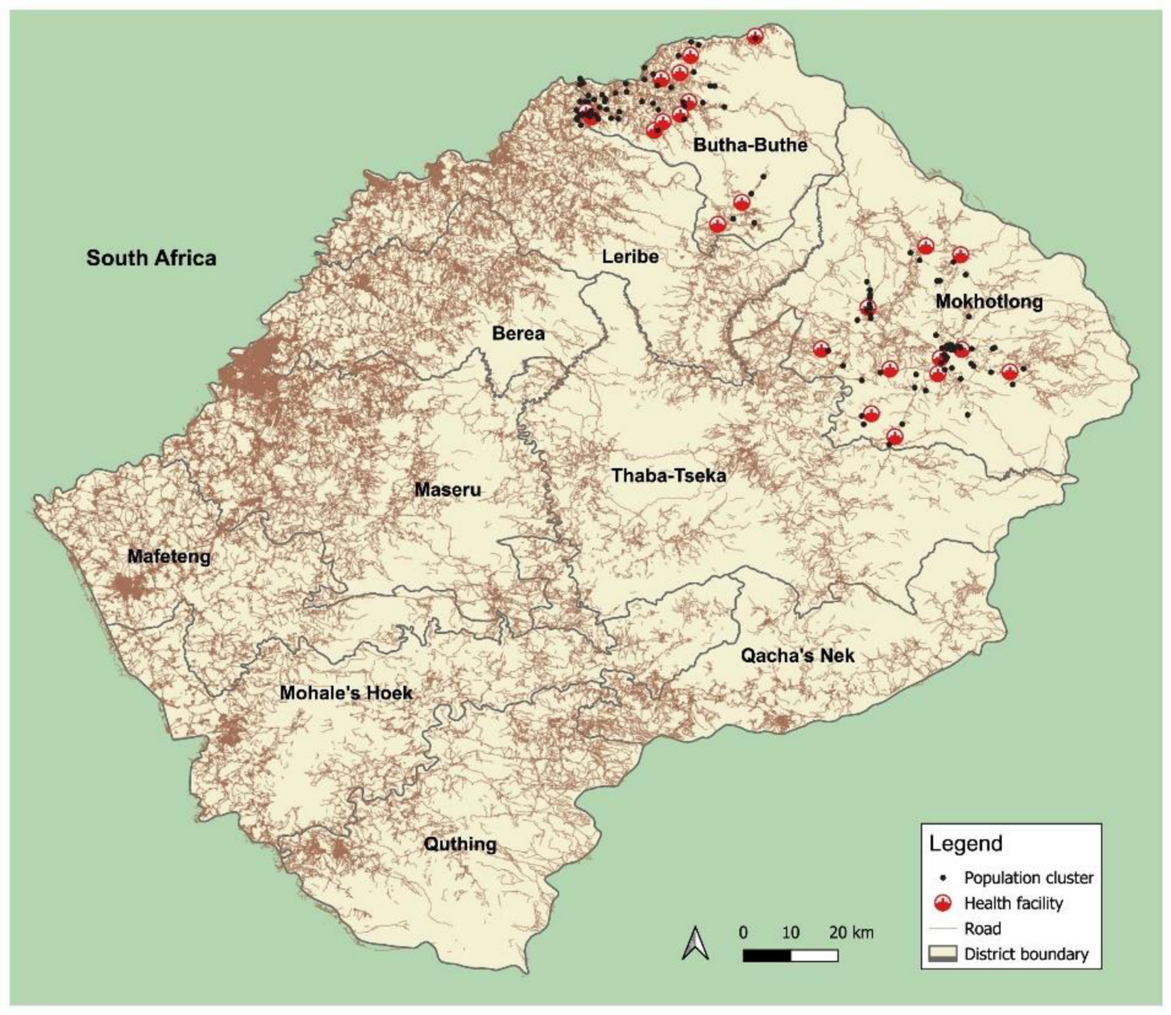
Map of Lesotho. The survey was conducted in Butha-Buthe and Mokhotlong districts, in the village clusters marked with black dots. Map produced by TL using QGIS v3.34 (QGIS.org. QGIS Geographic Information System. Open Source Geospatial Foundation Project. 2024; Available from: http://qgis.org).

This is a nested cross-sectional study during a large household-based survey among the general population in two districts in Northern Lesotho to assess the prevalence and determinants of several chronic diseases.^18^ Butha-Buthe and Mokhotlong districts have an estimated population of about 118 000 and 101 000, respectively. There is an adult HIV prevalence of 18.8% and 18.9% in Butha-Buthe and Mokhotlong, respectively, compared with the national prevalence of 22.7%.^19 20^ Each district has one central mid-size town, Butha-Buthe with ca. 25 000 inhabitants and Mokhotlong with ca. 10 000 inhabitants. The remaining population lives in rural villages scattered over a mountainous area of 5842 km^2^, predominantly subsistence farmers, mine workers, as well as construction or domestic labourers who work in neighbouring South Africa. Butha-Buthe has 10 nurse-led rural health centres, one missionary hospital, and one government hospital. In Mokhotlong, there are nine nurse-led rural health centres and one government hospital. Figure 1 displays a map of the country, including the village clusters involved in this study.

This study follows the Strengthening the Reporting of Observational Studies in Epidemiology reporting guidelines.^21^ A detailed description of the overall survey, where this cross-sectional study was embedded, has been published elsewhere.^18^ Briefly, a two-stage cluster sampling was used with the population clusters as primary sampling units and household members as secondary sampling units. From a list containing 785 clusters with at least 30 households each, 120 clusters were randomly selected (60 in each district), stratified by settlement (urban vs rural) and accessibility with respect to a catchment health facility (hard-to-reach vs easy-to-reach areas). All households in a sampled cluster were eligible if consent was provided by the head of household. Household members≥10 years old were randomly selected by an algorithm based on age, sex and settlement (rural vs urban), programmed in the Open Data Kit data collection tool.^22^ Adult participants provided written informed consent, if illiterate, consent documentation was sought by thumbprint and a witness had to provide a written consent. For participants below 18 years of age, a caregiver provided written consent and from the child verbal assent was sought. The sampled individuals were offered testing.

### Procedure and measurements

The study was conducted from 2 November 2021 to 31 August 2022. The study staff administered a detailed questionnaire to the randomly selected and consenting household members, collecting sociodemographic and medical history data. We used the Demographic and Health Survey programme questionnaire for Lesotho to calculate a wealth index for every household. The wealth index questionnaire assessed housing construction characteristics, household assets and utility services, including country-specific assets that are viewed as indicators of economic status in Lesotho^18 23^ and categorised into five quintiles ranging from poorest to wealthiest.^23^

Following the administration of questionnaires, the study staff then proceeded with hepatitis B surface antigen screening. We used the point-of-care Determine hepatitis B surface antigen (HBsAg2) test kit manufactured by Abbott Diagnostics Medical (Lake County, Illinois, USA).^24^ From participants who tested HBsAg-positive, a total of 0.25 mL of venous blood was collected on the spot. One drop (0.05 mL) of blood was applied to each of the five dried blood spot (DBS) card slots, using Whatman 903 cards.^25^ DBS cards were air-dried overnight, packaged according to instructions from the DBS card manufacturer and transported to the laboratory in Butha-Buthe where they were stored at −80 °C. Participants with a reactive HBsAg test were referred to one of the two nearest hospitals for a clinical follow-up assessment.

### Clinical follow-up assessment at the hospital

Dedicated study physicians conducted the clinical follow-up assessment of all participants who tested positive for HBsAg and who were linked to the hospital. The physicians assessed risk factors of HBV infection, constitutional symptoms and liver-specific symptoms and signs, according to the WHO Guidelines for the Prevention, Care and Treatment of persons with chronic hepatitis B infection.^26^ These risk factors included occupational risk factors such as being a health worker, a handler of hospital waste, involved in body grooming, or a sex worker; as well as having multiple sexual partners, recipient of blood transfusions, having major surgery, having body tattoos or traditional body markings. Assessed symptoms and signs included right hypochondrial pain, jaundice, body itch, dark urine, pale stool, fever, tremors, abdominal tenderness. Afterward, the study physicians drew venous blood for a complete blood count and liver function tests. Participants were treated according to the Standard Treatment Guidelines for Lesotho (2017).^27^

### Quantification of HBV DNA

The stored DBS samples were eventually transported to Switzerland and analysed for HBV viral load at the Kantonsspital Aarau using its laboratory’s standard operating procedure for analysis of DBS samples. The DBS samples were punched out from the filter paper and placed in an Eppendorf tube using tweezers. We then added 1.3 mL of a lytic reagent (guanidine thiocyanate) and vortexed well. During the following 2 hours, we vortexed occasionally until all the blood was dissolved from the filter paper. Finally, HBV quantification was performed on the Roche Cobas 5800 (Roche Diagnostics, Rotkreuz, Zug, Switzerland).

### Study outcomes

HBV infection was defined as having a positive HBsAg test result. Eligibility for HBV treatment was determined based on the 2024 WHO treatment guidelines^26^: (1) clinical evidence of compensated or decompensated cirrhosis, or Aspartate aminotransferase (AST) to Platelet Ratio Index (APRI) score>0.5; or (2) alanine aminotransferase (ALT) levels above 30 IU/L for men/above 19 IU/L for women and HBV DNA levels>2000 IU/mL; or (3) presence of HIV co-infection, family history of liver cirrhosis/cancer.

APRI score was calculated using the formula: ((AST in IU/L) / (AST upper limit of normal in IU/L) / (platelets in 10^9^/L)) × 100. The liver architecture was considered normal with an APRI<0.5; considered to have moderate to significant fibrosis with an APRI 0.5–2.0; and APRI>2 was considered suggestive of liver cirrhosis.^26 28 29^ HBV load was categorised to <2000, and >2000 to reflect low-level and high-level replication requiring treatment according to the 2024 WHO treatment guidelines.^26^

### Data collection and statistical analysis

Data was collected using KoboCollect (https://www.kobotoolbox.org/). Statistical analyses were conducted in Stata (V.16.1, StataCorp, College Station, Texas, USA). Descriptive statistics such as mean and SD; median and IQR were used for continuous variables, while frequency and percentage were used for categorical variables. The Ministry of Health of Lesotho introduced HBV vaccination at birth in 2003, that is, 18 years prior to our study. We therefore analysed age categorically as <18 years old, and ≥18 years old, to describe prevalence estimates separately in these two interesting groups. We used a multivariable logistic regression model to assess the factors associated with HBV infection. Variables with a p value<0.25 in univariate logistic regression were included in the final multivariable model.

Relationships between HBV viral load categories and age, sex, HIV status and antiretroviral therapy (ART) status were examined using cross-tabulation and visualised using bar plots. We tested for independence using the χ^2^ test.

### Patient and public involvement

This survey is part of the Community-based chronic care project Lesotho (ComBaCaL; www.combacal.org). It was designed together with the ComBaCaL steering committee that includes a community representative, as well as representatives from the Ministry of Health of Lesotho. The survey was discussed with local authorities (village chiefs, local Ministry of Health), who were engaged throughout the survey.

## Results

**Figure 2:**
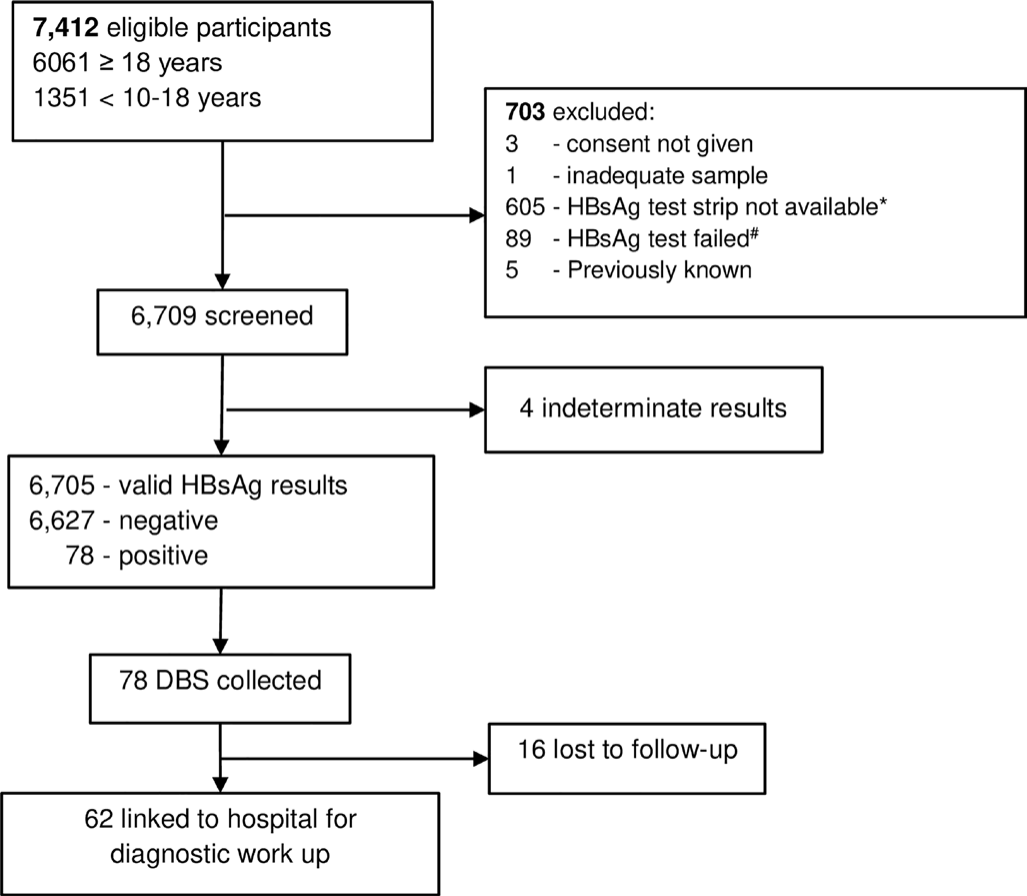
Study flow chart. DBS, dried blood spot; HBsAg, hepatitis B surface antigen. *Due to logistical reasons at the start of the survey, some HBsAg test strips were not available then. #These failed because of the point-of-care device.

Figure 2 shows the study flow. Among 7412 eligible participants, 6709 (90.5%) were screened for HBsAg and 6705 had a valid result. Of the 7412 eligible participants, 694 (9.4%) participants could not be screened due to logistical reasons. Overall, 78 had a reactive HBsAg test, yielding a point prevalence of 1.2% (95% CI: 0.9 to 1.4). Prevalence was lower among participants less than 18 years of age (0.24% (95% CI: 0.08 to 0.73)) compared with adult participants (1.4% (95% CI: 1.1 to 1.7)). Among the 78 participants screened HBsAg positive 62 attended the hospital for clinical assessment. Thus, DBS results are available from all 78 but detailed clinical evaluation and follow-up information only from 62/78 (79.5%) participants.

Table 1 displays the participants’ characteristics stratified by HBsAg status. The overall median age of participants was 33 years (IQR: 20–53), 1266/6705 (18.9%) were less than 18 years old, and 3509/6705 (52.3%) were female. The most recently published demographic health survey for Lesotho (2014) reports that the proportion of women in Lesotho was 53%, and 23.6% of the population was between 10 and 19 years old.^30^ This suggests that age and sex distribution were similar to the most recent demographic health survey in Lesotho. Among our participants, 838/6705 (12.5%) reported to be living with HIV. The median age among participants who tested positive for HBsAg was 45 years (IQR: 34–57), and 32 years (IQR: 20–53) for those who tested negative. Among participants who tested positive for HBsAg, 75/78 (96.1%) were≥18 years old, 49/78 (62.8%) were males, 58/78 (74.4%) lived in urban areas, and 26/78 (33.3%) were living with HIV.

**Table 1:** Characteristics of participants according to HBsAg status

Variable	HBsAg status		TotalN=6705
	Positiven=78	Negativen=6627	
Age, years (IQR)	45 (34–57)	32 (20–53)	33 (20–53)
Age categories			
<18	3 (3.9)	1263 (19.1)	1266 (18.9)
≥18	75 (96.1)	5364 (80.9)	5439 (81.1)
Sex			
Female	29 (37.2)	3480 (52.5)	3509 (52.3)
Male	49 (62.8)	3147 (47.5)	3196 (47.7)
Marital status*			
Single	16 (20.8)	1435 (25.4)	1451 (25.3)
In a committed relationship	44 (57.1)	3165 (55.9)	3209 (55.9)
Separated/divorced/widowed	17 (22.1)	1061 (18.7)	1078 (18.8)
BMI†			
<18.5	6 (7.8)	1109 (16.9)	1115 (16.8)
18.5–24.9	40 (51.9)	3264 (49.6)	3304 (49.7)
25–29.9	22 (28.6)	1204 (18.3)	1226 (18.4)
≥30	9 (11.7)	998 (15.2)	1007 (15.1)
Settlement			
Rural	20 (25.6)	3142 (47.4)	3162 (47.2)
Urban	58 (74.4)	3485 (52.6)	3543 (52.8)
Education			
None	9 (11.5)	542 (8.2)	551 (8.2)
Primary	36 (46.2)	3252 (49.1)	3288 (49.0)
Secondary	24 (30.8)	2430 (36.7)	2454 (36.6)
Tertiary	9 (11.5)	403 (6.1)	412 (6.1)
Employment‡			
Unemployed	33 (42.3)	3817 (57.8)	3850 (57.6)
Employed	45 (57.7)	2786 (42.2)	2831 (42.4)
HIV status			
Positive	26 (33.3)	812 (12.3)	838 (12.5)
Negative	52 (66.7)	5815 (87.8)	5867 (87.5)
Wealth index§			
First quintile	7 (9.0)	1310 (19.8)	1317 (19.7)
Second quintile	11 (14.1)	1312 (19.9)	1323 (19.8)
Third quintile	18 (23.1)	1315 (19.9)	1333 (19.9)
Fourth quintile	19 (24.4)	1318 (19.9)	1337 (20.0)
Fifth quintile	23 (29.5)	1354 (20.5)	1377 (20.6)
Lifestyle			
Tobacco consumption¶††			
Yes	31 (39.7)	1386 (21.0)	1417 (21.2)
No	47 (60.3)	5231 (79.1)	5278 (78.8)
Alcohol consumption**††			
Yes	25 (32.1)	1521 (23.0)	1546 (23.1)
No	53 (67.9)	5096 (77.0)	5149 (76.9)
Intravenous drug use‡‡			
Yes	3 (3.9)	257 (3.9)	260 (3.9)
No	75 (96.1)	6370 (96.1)	6445 (96.1)

* 966 missing marital status data among participants HBsAg negative, 1one missing among HBsAg positive.

† 52 missing BMI data among participants HBsAg negative, 1one missing among those positive.

‡ 24 missing employment data among participants HBsAg negative.

§ 18 missing wealth index data among participants HBsAg negative. FThe first quintile is the poorest, fifth quintile the richest.

¶ 10 missing tobacco consumption data among participants HBsAg negative.

** 10 missing alcohol consumption data among participants HBsAg negative.

†† In the 3 months prior.

‡‡ Any history.

BMIbody mass indexHBsAgHBV surface antigen

Results of univariate and multivariable logistic regression models for HBsAg positivity are given in table 2. Being≥18 years old (adjusted OR (aOR), 4.29; 95% CI: 1.27 to 14.43; p=0.019), male (aOR, 1.88; 95% CI: 1.11 to 3.19; p=0.018), living in urban areas (aOR, 2.54; 95% CI: 1.44 to 4.48; p=0.001), living with HIV (aOR, 3.09; 95% CI: 1.88 to 5.08; p<0.001) and consuming tobacco (aOR, 2.17; 95% CI: 1.23 to 3.82; p=0.007) were associated with increasing odds of HBsAg positivity. Compared with participants within the first (lowest) wealth index quintile, participants in the third, fourth and fifth quintiles had significantly higher odds of HBsAg diagnosis.

**Table 2:** Univariable and multivariable logistic regression models of factors associated with seropositivity

Variable	n (%)	Univariate analysis		Multivariable analysis	
		OR (95% CI)	P value	aOR(95% CI)	P value
Age categories					
<18 years old	3 (3.9)	1	–	1	–
≥18 years old	75 (96.1)	5.89 (1.85 to 18.70)	0.003	4.29 (1.27 to 14.43)	0.019
Sex					
Female	29 (37.2)	1		1	–
Male	49 (62.8)	1.87 (1.18 to 2.96)	0.008	1.88 (1.11 to 3.19)	0.018
Marital status					
Single	16 (20.8)	1			
In a committed relationship	44 (57.1)	1.25 (0.70 to 2.22)	0.453		
Separated/divorced/widowed	17 (22.1)	1.44 (0.72 to 2.86)	0.301		
Settlement					
Rural	20 (25.6)	1		1	–
Urban	58 (74.4)	2.61 (1.57 to 4.36)	<0.001	2.54 (1.44 to 4.48)	0.001
Education					
None	9 (11.5)	1		1	–
Primary	36 (46.2)	0.67 (0.32 to 1.39)	0.280	0.83 (0.39 to 1.76)	0.625
Secondary	24 (30.8)	0.59 (0.27 to 1.29)	0.187	0.69 (0.31 to 1.55)	0.367
Tertiary	9 (11.5)	1.34 (0.53 to 3.42)	0.534	1.17 (0.43 to 3.17)	0.753
Employment					
Unemployed	33 (42.3)	1	–	1	–
Employed	45 (57.7)	1.87 (1.19 to 2.94)	0.007	0.99 (0.61 to 1.62)	0.980
Reported HIV status					
Negative	52 (66.7)	1	–	1	–
Positive	26 (33.3)	3.58 (2.22 to 5.77)	<0.001	3.09 (1.88 to 5.08)	<0.001
Wealth index*					
First quintile	7 (9.0)	1	–	1	–
Second quintile	11 (14.1)	1.57 (0.61 to 4.05)	0.353	1.95 (0.75 to 5.10)	0.173
Third quintile	18 (23.1)	2.56 (1.07 to 6.15)	0.035	3.00 (1.22 to 7.15)	0.016
Fourth quintile	19 (24.4)	2.70 (1.13 to 6.44)	0.025	2.59 (1.07 to 6.28)	0.036
Fifth quintile	23 (29.5)	3.18 (1.36 to 7.43)	0.008	2.79 (1.15 to 6.75)	0.023
Tobacco consumption†					
No	47 (60.3)	1	–	1	–
Yes	31 (39.7)	2.49 (1.58 to 3.93)	<0.001	2.17 (1.23 to 3.82)	0.007
Alcohol consumption†					
No	53 (67.9)	1	–	1	–
Yes	25 (32.1)	1.58 (0.98 to 2.55)	0.061	0.66 (0.37 to 1.18)	0.157
Intravenous drug user‡					
No	75 (96.1)	1	–		
Yes	3 (3.9)	0.99 (0.31 to 3.16)	0.988		

* First quintile is the poorest, fifth quintile is the richest.

† in the 3 months months prior.

‡ Any history.

aOR, adjusted OR

For 56/78 (71.8%) participants, the HBV viral load was less than 2000 IU/mL, and for 22/78 (28.2%) participants, the viral load was more than 2000 IU/mL. Online supplemental figure S1 shows HBV viral load by sex, age and HIV status, and tenofovir/lamivudine-containing ART among those living with HIV. Those receiving ART had a significantly lower HBV viral load compared with those not receiving ART: of 23 participants on ART, 2/23 (8.7%) had HBV DNA>2000 IU/mL vs 16/39 (41.0%) among those not taking ART (p=0.007).

**Figure 3:**
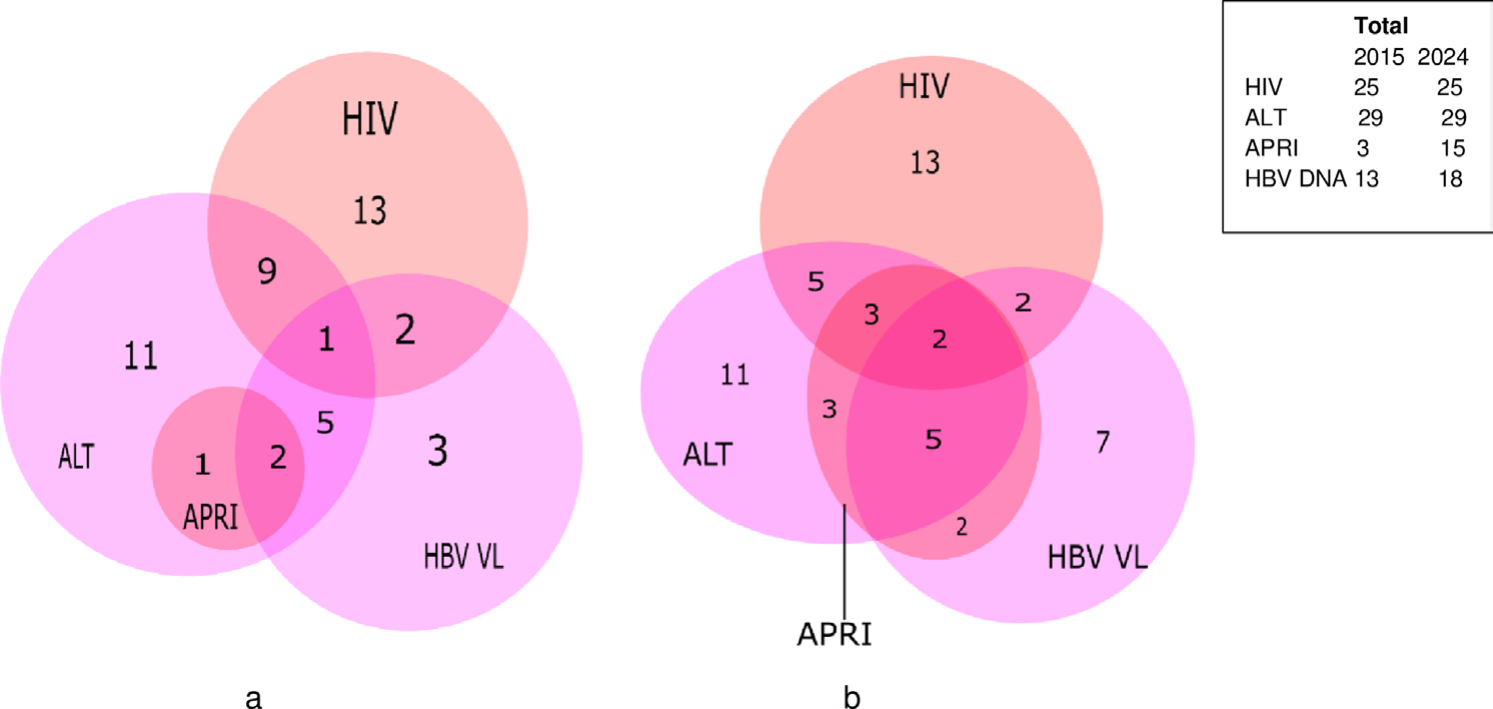
Venn diagram showing treatment eligibility among participants linked to care using (**a**) WHO 2015 guidelines, and (**b**) WHO 2024 guidelines. ALT, alanine aminotransferase; APRI, Aspartate aminotransferase to Platelet Ratio Index; HBV VL, hepatitis B virus viral load. (a) WHO guidelines 2015: Antiviral treatment eligibility fulfilled by either (i) APRI>2, or (ii) VL>20 000 IU/mL and ALT>30 IU/L for men/>19 IU/L for women, or (iii) HIV positive. Total eligible: 33/62 (53.2%). (b) WHO guidelines 2024: Antiviral treatment eligibility fulfilled by either (i) APRI>0.5, or (ii) VL>2000 IU/mL and ALT>30 IU/L for men/>19 IU/L for women, or (iii) HIV positive/family history of liver cirrhosis or cancer. Total eligible: 35/62 (56.5%).

Online supplemental table S1 summarises the characteristics of the 62 participants who tested HBsAg-positive who were linked to care and thus underwent a clinical and laboratory assessment. Among them, 35/62 (56.5%) fulfilled the WHO 2024 eligibility criteria for antiviral treatment. 25 (25/35, 71.4%) were eligible due to HIV co-infection (figure 3). 15 participants (15/62; 24.2%), had an APRI score>0.5. 18 participants (18/62, 29.0%) participants had HBV viral load levels above 2000 IU/mL, 29/62 (46.8%) participants had ALT levels above 30 U/L for males/above 19 IU/L for females. Among the 37 mono-infected participants, 10 (27%) were eligible for treatment.

In comparison, using the 2015 WHO guidelines,^31^ valid at the time of the study, to determine treatment eligibility (APRI score>2; or ALT levels above 30 IU/L for men/above 19 IU/L for women and HBV DNA levels>20 000 IU/mL; or presence of HIV co-infection), a total of 33/62 (53.2%) were eligible, 25/33 (75.8%) due to HIV co-infection. Three (3/62, 4.8%) had APRI score >2 and 13/62 (21.0%) had HBV viral load levels above 20 000 IU/mL.

Lowering the treatment threshold of the APRI score from 2 to 0.5 and HBV viral load from 20 000 to 2000 IU/mL in the 2024 WHO guidelines increases the number of eligible participants. However, in our sample, most participants were eligible due to co-infection with HIV and thus, overall, the total number of eligible participants only increased from 33/62 (53.2%; 2015 guidelines) to 35/62 (56.5%; 2024 guidelines) participants (figure 3).

## Discussion

This study determined the prevalence of HBV infection among the general population in two districts in Lesotho, Southern Africa, assessed the determinants of HBV infection, and treatment eligibility according to the 2024 WHO guidelines. HBV infection among Basotho was low, but substantially more prevalent among adults compared with younger participants. Male sex, urban settlement, presence of HIV and increasing household wealth were positive determinants of HBV infection. A high proportion of participants linked to care were eligible for antiviral treatment, based on the WHO treatment eligibility criteria. Participants on ART had significantly lower HBV DNA levels compared with participants not on ART. These findings offer insights into factors associated with the risk of HBV infection in Lesotho, suggest a testing and treatment gap especially among people not living with HIV, and emphasise the importance of ART to lower HBV DNA.

Our study addresses an important knowledge gap on the epidemiology of HBV infection in the general population in Lesotho, adding to the relevant health data aggregation, and informing policy and planning in the country. Until now, available data in the setting have focused on hospitalised patients and key populations such as people living with HIV.^16 17^ Recent Global Burden of Disease hepatitis B estimates for Southern Africa report a prevalence of 4.5% overall, 6.9% for Lesotho, 3.5% and 4.9% for neighbouring South Africa and Eswatini, respectively.^2^ Compared with these numbers, our study found a lower prevalence of HBV infection. A likely explanation for the disparity is that the Global Burden of Disease estimates are based on the only two publicly available studies on the prevalence of HBV infection in the country—conducted among people living with HIV, with a reported prevalence of HBV/HIV co-infection of 10.5%^17^ and 4.3%.^16^ Available data indicate that the Ministry of Health of Lesotho first rolled out the programme on hepatitis B vaccination at birth in 2003^11^ (18 years prior to this study). According to the 2023 WHO and UNICEF estimates for immunisation coverage for Lesotho, the percentage of infants who received the third dose of hepatitis B vaccine has ranged between 87% and 96% over the past decade.^32^ This certainly has contributed to the low HBV prevalence in the younger individuals included in this study.^33 34^ A recent study in Malawi reported an HBV infection prevalence of 0.3% among participants born after vaccine introduction^35^—similar to the 0.24% prevalence observed in our study population aged less than 18 years old.

Beyond age, the associations between male sex, urban settlement, presence of HIV and HBV infection have been documented in other studies.^36 37^ It has been suggested that men are more susceptible to HBV infection due to behavioural risk factors and the effects of androgen hormones.^36 38 39^ Interestingly, we found that the odds of HBV infection increased in higher wealth groups compared with the lowest wealth group. Studies conducted within and outside of Africa show that increasing wealth is associated with higher hepatitis B vaccine coverage, and lower HBV infection.^3540^,^42^ However, it has been reported that belonging to the richest income households is associated with having multiple sexual partners which may explain our findings.^43^,^45^

The reduced HBV DNA in people living with HIV and taking ART, compared with participants not taking ART shows the effect of tenofovir-based ARTs to suppress HBV replication.^46^,^49^ One participant who was not suppressed despite being on tenofovir-based ART could be attributed to non-adherence. In addition, we found a high proportion of HIV-negative individuals who were eligible for treatment (21.6%) but were untreated. From the literature, treatment eligibility of HIV-negative HBsAg-positive individuals varies considerably, ranging from 2% to 22% in the African region.^35 50 51^ In the absence of routine HBV viral load testing and FibroScan to assess liver architecture, WHO treatment eligibility guidelines are difficult to apply in a setting such as Lesotho. The general focus in these settings is on HIV, while mono-infection with HBV is a neglected health topic. The new 2024 WHO guidelines lower the treatment eligibility thresholds for APRI score and HBV DNA levels and therefore allow more people to be treated, however, the requirement to measure HBV viral load remains. In a setting such as Lesotho with high HIV prevalence, the number of participants eligible for treatment increases only marginally when applying the 2024 guidelines versus the 2015 guidelines. Our study shows that there is a substantial number of people living only with HBV and who are treatment eligible—mostly determined by HBV viral load. More research is needed to identify mono-infected participants and to simplify the treatment eligibility criteria for these individuals in order to reduce the treatment gap. Point-of-care HBV viral load and widespread rollout of HBV viral load testing have been suggested as a solution.^52 53^

Key strengths of our study are the large sample size, a systematic sampling of the study population and the fact that our sample was a general population rather than a specific key population subgroup. Our study has several limitations. First, because of the nature of the study, the chronicity of HBV infection could not be determined since tests could only be performed once. Second, some participants with HBV infection were lost to follow-up with the potential to introduce a selection bias in our evaluation for treatment eligibility. Third, a FibroScan would have been desirable as an additional treatment eligibility criterion but is not routinely available in Lesotho and was not feasible to introduce during the study period. Finally, for participants with HIV, data on HIV viral load nor duration of ART were not available to provide further insight into potential factors contributing to HBV treatment eligibility, efficacy or resistance.

In summary, we conducted a large-scale population-based study to determine the prevalence of HBV infection in a general population sample in two districts in Lesotho, and assess the determinants of HBV infection and treatment eligibility according to the 2024 WHO guidelines. HBV infection in the population was low, but higher among adults than younger participants, and positively associated with male sex, urban settlement, presence of HIV and increasing household wealth. Being on ART was associated with a significantly lower HBV DNA level. Our study found a significant testing and treatment gap among individuals not living with HIV, who need antiviral treatment. Increased efforts and research are needed to identify and treat people with hepatitis B but without HIV, and simplified treatment eligibility criteria for settings such as Lesotho would be desired.

## supplementary material

10.1136/bmjph-2024-001195 online supplemental file 1

## Data Availability

Data are available upon reasonable request.
