# Supplementary material for: Prevalence of hepatitis B virus infection and treatment eligibility in Lesotho, Southern Africa: a population-based cross-sectional study with case-based follow-up
Source: BMJ Public Health. 2024 Nov 29;2(2):e001195. doi: 10.1136/bmjph-2024-001195 (PMC11816210; doi:10.1136/bmjph-2024-001195)
Supplement: online supplemental file 1 [file bmjph-2-2-s001.pdf]

Prevalence of hepatitis B virus infection and treatment eligibility in Lesotho, Southern Africa

Emmanuel Firima *PhD*[1,2*], Rameno Ntsoaki *MBBS*[3], Blaise Lukau *MBBS*[4], Mosa Tlahali *MBBS*[5], Lucia Gonzalez *PhD*[1], Molulela Manthabiseng *BNSc*[4], Mamoronts'sane P. Sematle *BNSc*[4], Matumaole Bane *BNSc*[4], Makhebe Khomolishoele *BNSc*[4], Ikhetheleng Leisa *BScNM*[4], Lefokotsane Retselisitsoe *BScNM*[4], Ravi Gupta *MBBS*[4], Stephen McCrosky *MSN*[1], Tristan Lee *MSc*[1,6], Frédérique Chammartin *PhD*[1], Maja Weisser *MD*[7,8], Niklaus Daniel Labhardt *MD*[1#] and Alain Amstutz *PhD*[1,9,10#]

1. Division of Clinical Epidemiology, Department of Clinical Research, University Hospital Basel, University of Basel, Basel, Switzerland
2. Division of Clinical Medicine, School of Medicine and Population Health, University of Sheffield, Sheffield, United Kingdom
3. Butha-Buthe Government Hospital, Butha-Buthe, Lesotho
4. SolidarMed, Partnerships for Health, Maseru, Lesotho
5. Mokhotlong District Health Management Team, Mokhotlong, Lesotho
6. Swiss Tropical and Public Health Institute, Allschwil, Switzerland
7. Division of Infectious Diseases and Hospital Epidemiology, University Hospital Basel, Basel, Switzerland
8. Ifakara Health Institute, Ifakara, Tanzania
9. Population Health Sciences, Bristol Medical School, University of Bristol, Bristol, UK
10. Oslo Center for Biostatistics and Epidemiology, Oslo University Hospital, Oslo, Norway

[#] The authors contributed equally to the manuscript and share last-authorship position

***Correspondence to**: Emmanuel Firima, Division of Clinical Epidemiology, University Hospital and University of Basel, Totengässlein 3, 4051 Basel, Switzerland. emanfirima@gmail.com

Supplementary Material

S1 figure. Hepatitis B viral load results across (a) sex; (b) age categories; (c) HIV status; (d) ART status.

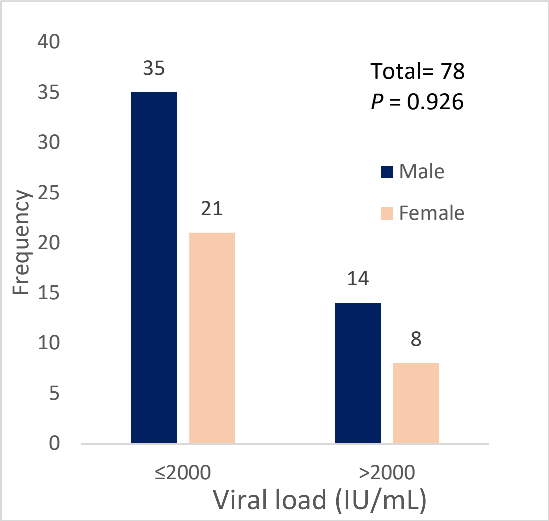

(a) Sex

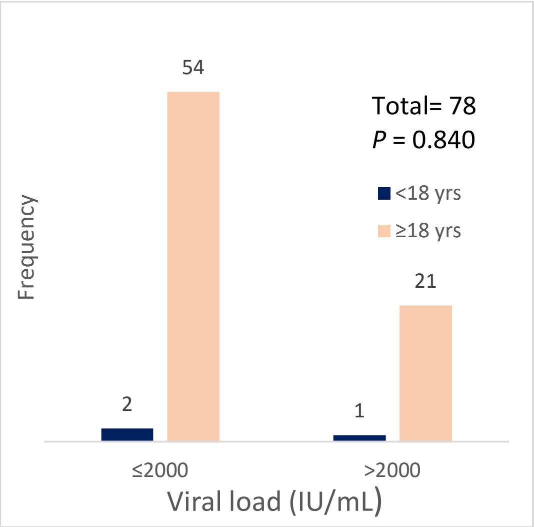

(b) Age

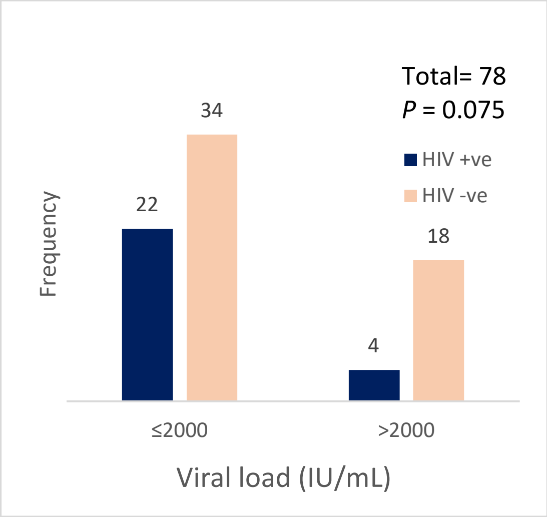

(b) HIV status

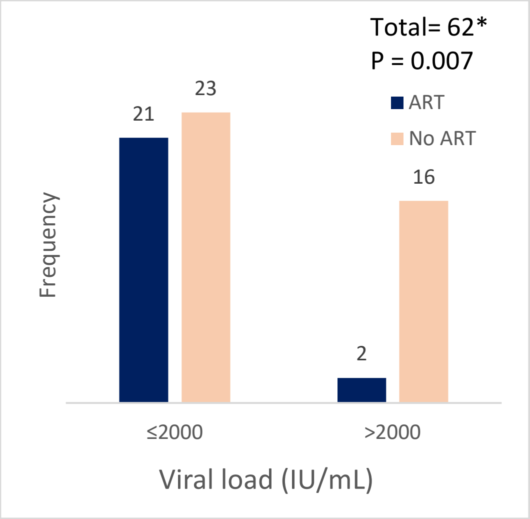

(d) ART status

ART: anti-retroviral therapy; yrs: years; +ve: positive; -ve: negative; *ART assessed among those linked to care. Of the 2 on ART and with viral load > 2000 IU/mL, 1 was on abacavir-based regimen.

S1 table. Characteristics of participants with a positive HBsAg test who were linked to care, stratified by treatment eligibility.

**Variable**	**Total N=62 n(%)**	**Treatment eligible N= 35 n(%)**	**Treatment ineligible N=27 n(%)**
Occupational risk group[a]			
No	57 (91.9)	32(91.4)	25(92.6)
Yes	5 (8.1)	3(8.6)	2(7.4)
Liver-related symptoms[b]			
Absent	38 (61.3)	19(54.3)	19(70.4)
Present	24(38.7)	16(45.7)	8(29.6)
Liver-related signs[b]			
Absent	38(61.3)	20(57.1)	18(66.7)
Present	24(38.7)	15(42.9)	9(33.3)
HIV status			
negative	37(59.7)	10(28.6)	27(100)
positive	25(40.3)	25(71.4)	0(0)
Taking ART[c]			
No	39(62.9)	12(34.3)	27(100)
Yes	23(37.1)	23(65.7)	0(0)
Other self-reported sexually transmitted diseases (current or previous)			
No	52 (83.9)	28(80.0)	24(88.9)
Yes	10(16.1)	7(20.0)	3(11.1)
Any blood transfusion in the past			
No	58(93.5)	32(91.4)	26(96.3)
Yes	4(6.5)	3(8.6)	1(3.7)
Any major surgery in the past			
No	54(87.1)	32(91.4)	22(81.5)
Yes	8(12.9)	3(8.6)	5(18.5)
Any body tattoos			
No	57(91.9)	31(88.6)	26(96.3)
Yes	5(8.1)	4(11.4)	1(3.7)
Any traditional bodily markings			
No	37(59.7)	20(57.1)	17(63.0)
Yes	25(40.3)	15(42.9)	10(37.0)
Multiple sexual partners			
No	50(80.6)	27(77.1)	23(85.2)
Yes	12(19.4)	8(22.9)	4(14.8)
Blood tests, median (IQR)			
AST, IU/L	33(23-43)	36(28-49)	29(18-33)
ALT, IU/L	26(18-34)	29(19-43)	19(14-31)
ALT > ULN, %	29(46.8)	19(54.3)	10(37.0)
ALT ≤ ULN, %	33(53.2)	16(45.7)	17(63.0)
Bilirubin, mg/dL	1(0-7.4)	1.9(0-9.3)	0(0-6.9)
alkaline phosphatase, IU/L	95(78-128)	95(83-128)	93.5(57-121)
gamma glutamyl transaminase, IU/L	23(16-42.2)	27(17-47)	20(12-27)

platelet count, x1000 cells/ µL	234(197-275)	210(167-268)	245(217-342)
Haemoglobin, g/dL	15.1(13.6-16.3)	15.3(13.8-16.4)	14.8(12.6-16.2)
white blood cells, x1000 cells/ µL	5.6(4.3-6.9)	5.5(4.1-6.9)	5.9(4.8-7.2)
APRI score[d]			
Median (IQR)	0.4(0.2-0.5)	0.4(0.3-0.7)	0.3(0.2-0.4)
> 0.5	15(24.2.6)	15(42.9)	0(0)
≤ 0.5	42(75.8)	19(57.1)	23(100)
HBV DNA[e], IU/mL			
> 2000	18(29.0)	11(31.4)	7(25.9)
≤ 2000	44(71.0)	24(68.6)	20(74.1)

ART: antiretroviral therapy; IQR: interquartile range; AST: aspartate aminotransferase; ALT: alanine aminotransferase; ULN: upper limit of normal (*> 30 IU/L for males/ > 19 IU/L for females*); IU/L : international units per liter; mg/dL : miligrams per deciliter; µL : microliter; g/dL: grams per deciliter; APRI: aspartate aminotransferase to platelet ratio index;

a Occupational risk factors included being a health worker, a handler of hospital waste, involved in body grooming, or sex worker.

b Symptoms and signs of liver diseases included right hypochondrial pain, jaundice, body itch, dark urine, pale stool, fever, tremors, abdominal tenderness;

c 3 were on abacavir-based regimen.

d 5 missing APRI values.

e 78 participants had HBV DNA result, but only results for the 62 participants who were linked to care shown in this table.
